# TIP peptide inhalation in oleic acid-induced experimental lung injury: a post-hoc comparison

**DOI:** 10.1186/1756-0500-6-385

**Published:** 2013-09-27

**Authors:** Erik K Hartmann, Alexander Bentley, Bastian Duenges, Klaus U Klein, Stefan Boehme, Klaus Markstaller, Matthias David

**Affiliations:** 1 Department of Anaesthesiology, Medical Centre of the Johannes Gutenberg-University, Langenbeckstr. 1, 55131 Mainz, Germany; 2 Department of Anaesthesia, General Critical Care Medicine and Pain Therapy, Medical University of Vienna, Waehringer Guertel 18-20, 1090 Vienna, Austria

**Keywords:** Lung injury, Pulmonary edema, TIP peptide, Lectin-like domain, Porcine model

## Abstract

**Background:**

The lectin-like domain of TNF-α mimicked by an inhaled TIP peptide represents a novel approach to attenuate a pulmonary edema in respiratory failure, which is on the threshold to clinical application. In extension to a previously published study, which reported an improved pulmonary function following TIP peptide inhalation in a porcine model of lavage-induced lung injury, a post-hoc comparison to additional experiments was conducted. This analysis addresses the hypothesis that oleic acid injection-induced capillary leakage and alveolar necrosis blunts the previously reported beneficial effects of TIP peptide inhalation in a porcine model.

**Findings:**

Following animal care committee approval lung injury was induced by oleic acid injection in six pigs with a setting strictly according to a previously published protocol that was used for lung-lavaged pigs. Ventilation/perfusion-distribution by multiple inert gas elimination, parameters of gas exchange and pulmonary edema were assessed as surrogates of the pulmonary function. A significantly improved ventilation/perfusion-distribution following TIP inhalation was recognized only in the bronchoalveolar lavage model but not following oleic acid injection. The time course after oleic acid injection yielded no comparable impact of the TIP peptide on gas exchange and edema formation.

**Conclusions:**

Reported beneficial effects of the TIP peptide on gas exchange and pulmonary edema were not reproducible in the oleic acid injection model. This analysis assumes that sustained alveolar epithelial necrosis as induced by oleic acid injection may inhibit the TIP-induced edema resolution. Regarding the on-going clinical development of the TIP peptide this approach should hardly be effective in states of severe alveolar epithelial damage.

## Findings

### Background

In extension to a previously published study [1] a post-hoc comparison to additionally acquired data was performed. The lectin-like domain of TNF-α mimicked by an inhaled TIP peptide is a novel pharmacologic approach for treatment of edematous respiratory failure and acute respiratory distress syndrome (ARDS) [2]. In several experimental models TIP peptides were shown to attenuate pulmonary edema formation [3-6]. The TIP peptide primarily stimulates epithelial sodium channels on type II alveolar cells to enhance sodium transfer across the alveolar epithelium. This generates an osmotic gradient and allows the absorption of an alveolar edema, whereas this effect can be inhibited by pharmacologic blockade of the epithelial sodium channels [3-5]. Furthermore, the TIP peptide reduces microvascular hyperpermeability, which prevents a further edema formation [4,7], and was found to reduce inflammatory response and reactive oxygen species generation in rat model of ischemia and reperfusion-related lung injury [6]. Despite proceeding to the early clinical study phase only few data of TIP peptide application are available from clinical-like in vivo models. Our group found a significantly improved pulmonary function when an inhaled TIP peptide was compared to placebo in a porcine model of lung lavage/surfactant-depletion (LAV)-induced lung injury [1]. This finding was primarily attributed to resolution of pulmonary edema through the TIP peptide as driven by epithelial sodium channels and the basolateral Na^+^-K^+^-ATPase in the intact alveolar epithelium. Hence, an intact alveolar epithelium should be critical for the efficiency and clinical benefit of the TIP peptide in an in vivo setting. Oleic acid injection (OAI) is another proven ARDS model that is primarily based on inducing capillary leakage and epithelial necrosis [8].

We hypothesized that beneficial effect of the TIP peptide would be blunted by OAI. A post-hoc analysis therefore compared lung injured pigs by OAI to a previous study [1].

### Material and methods

Following approval of the state and institutional animal care committee (Landesuntersuchungsamt Rheinland-Pfalz, Koblenz, Germany) six pigs were prospectively examined and post-hoc compared to six randomly selected animals with LAV-induced lung injury to provide further insights of model-dependent characteristics of the TIP peptide.

Anesthesia, experimental procedure, monitoring and ventilator settings were strictly adapted to the previous study. The OAI group (n = 6) received an intravenous application of oleic acid solved in balanced electrolyte solution in a ratio of 1:10. The oleic acid was then applied in fractions of 1–2 ml over thirty minutes. Short-term hemodynamic instability, which regularly occurs immediately after injection, was treated by norepinephrine boli of 5–10 μg. To provide comparable baseline conditions the procedure was continued until the quotient of arterial partial pressure of oxygen and inspired oxygen fraction was < 250 mmHg over 30 minutes or a dose maximum of 0.3 ml kg^-1^ was administered. Starting with the lung injury induction, all animals were ventilated in a constant mode that aims to avoid lung protective effects: tidal volume 10 ml kg^-1^, positive end-expiratory pressure 0 cmH_2_O, fraction of inspired oxygen 1.0 and respiratory frequency 25–35 min^-1^ targeted to an end-tidal carbon dioxide level < 60 mmHg. The TIP peptide (1 mg kg^-1^; AP301, APEPTICO, Vienna, Austria) was prepared and administered by nebulization once stable ARDS conditions were achieved. The animals were monitored over three hours after TIP peptide inhalation. The ventilation/perfusion-distribution (V_A_/Q) measured by micropore membrane inlet mass spectrometry – multiple inert gas elimination (MMIMS-MIGET, Oscillogy LLC, PA, USA) was assessed as main parameter in a previously described manner [9,10]. V_A_/Q data was also available from the previous study. Additionally, parameters of gas exchange, extravascular lung water by transpulmonary thermodilution and respiratory mechanics (dynamic lung compliance) were examined. At the end of the protocol the animals were killed in deep general anesthesia by central venous potassium injection (20 mval).

Parameters are given as median and interquartile range. Intergroup comparison of the ventilation/perfusion-distribution was conducted by the Mann–Whitney-U-Test. Time courses of main parameters from the OAI group were analyzed by Friedman-Test with post-hoc Dunn-Test. P values lower than 0.05 were regarded as different. Comparisons between the previously published study at baseline and after three hours and the OAI group were drawn at baseline and after three hours by non-parametric testing in explorative manner with descriptive P values (Table 1).

**Table 1:** Study data

**Parameter**	**ARDS-baseline**		**3 hours**	
	**OAI**	**LAV**[1]	**OAI**	**LAV [**[1]**]**
paO_2_/FiO_2_ [mmHg]	172 (91)	157 (59)	203 (151)	260 (63)*
EVLWI [ml kg^-1^]	14 (4)	15 (2)	13 (2)	13 (1)*
C_dyn_[ml cmH_2_O^-1^]	16 (2)	15 (3)	13 (3)*	17 (4)
V_t_ [ml kg^-1^]	9.9 (0.5)	10.0 (0.2)	10.2 (0.5)	9.8 (1.4)
PEEP [cmH_2_O]	0.6 (0.1)	0.5 (0.1)	0.7 (0.6)	0.7 (0.4)
RR [min^-1^]	27 (3)	25 (4)	27 (4)	26 (4)
P_endinsp_ [cmH_2_O]	16 (2)	19 (4)	20 (4)	18 (2)
FiO_2_	1.0	1.0	1.0	1.0
etCO_2_ [mmHg]	43 (4)	38 (4)	36 (2)	37 (4)
PaCO_2_ [mmHg]	68 (12)	59 (9)	48 (18)	45 (4)
MAP [mmHg]	97(13)	108 (15)	82 (5)	86 (16)
MPAP [mmHg]	45 (7)	20 (7) #	31 (5)*	19 (5) #
CO [l min^-1^]	3.9 (0.2)	4.0 (1.0)	3.4 (0.3)	3.6 (0.5)
PVR [dyn s cm^-5^]	584 (265)	187 (158) #	349 (175)*	183 (124) #

Data expressed as median (interquartile range). * indicates P < 0.05 vs. ARDS baseline value, # indicates P < 0.05 in intergroup comparison vs. OAI.

### Results and conclusions

**Figure 1:**
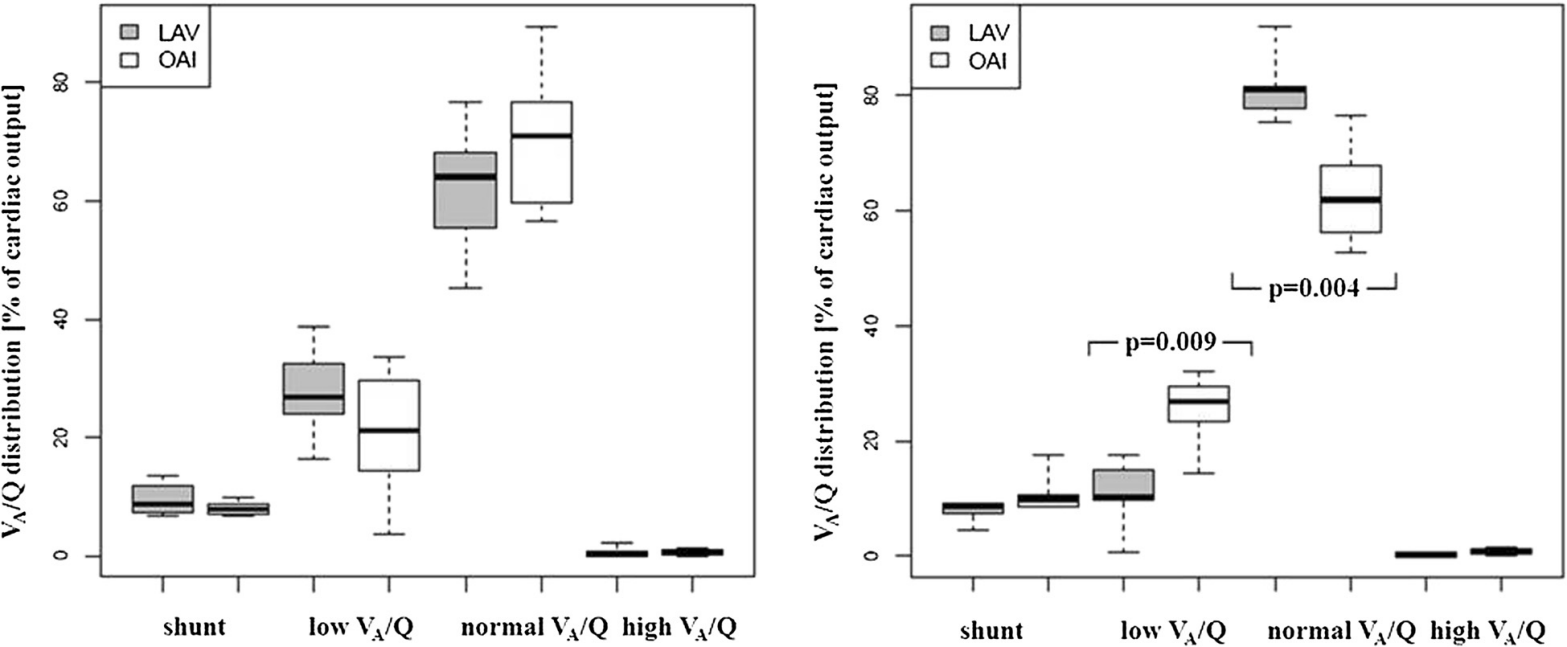
**Ventilation/perfusion-distribution (V**_**A**_**/Q) in both groups.** Comparable baseline conditions (left) and significantly improved V_A_/Q in the LAV group within three hours (right).

**Figure 2:**
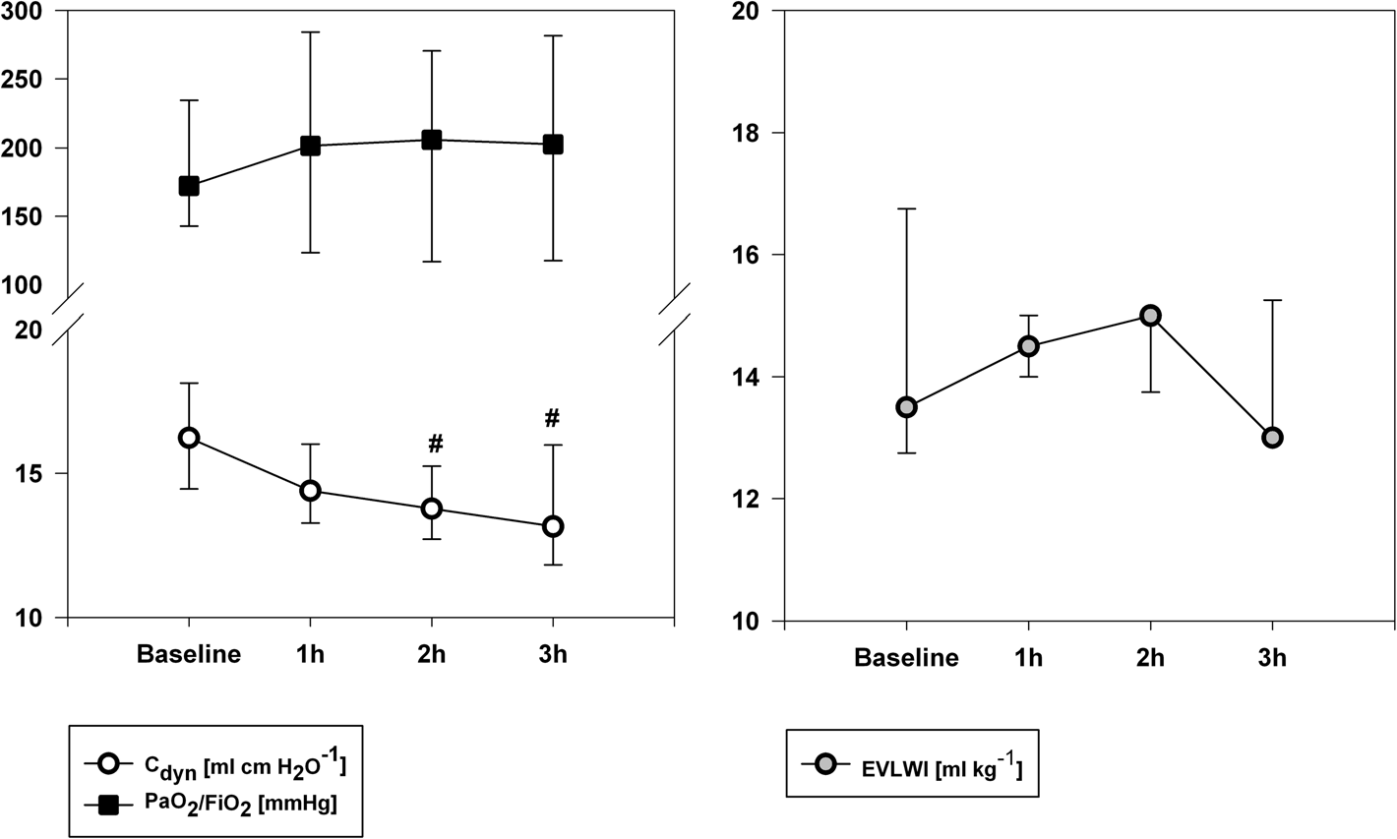
**Time courses of oxygenation (PaO**_**2**_**/FiO**_**2**_**), dynamic lung compliance (C**_**dyn**_**) and extravascular lung water content (EVLWI).** # indicates P < 0.05 vs. baseline value.

Comparable baseline conditions for the two models (LAV, OAI) were achieved in terms of V_A_/Q, gas exchange, pulmonary edema. Hemodynamics remained stable without vasopressor support over three hours in both groups. Ventilator settings and hemodynamics showed no relevant differences other than higher values of mean pulmonary arterial pressure and pulmonary vascular resistance in the OAI group, which can be explained by different model features respectively specific characteristics of OAI (Table 1). Within three hours after TIP inhalation the overall lung function as measured by V_A_/Q distribution approved a significantly higher fraction of normal ratios (P = 0.004 vs. OAI group) and a lower amount a poorly ventilated lung areas (low V_A_/Q, P = 0.009 vs. OAI group; Figure 1) in the LAV group. As previously reported [1] the LAV group showed an increase of oxygenation and decrease of extravascular lung water content after TIP inhalation (Table 1). These findings were not reproducible in the OAI group (Figure 2). In the OAI group an on-going worsening of dynamic lung compliance was also registered (Figure 2). Despite the improved V_A_/Q in the LAV group and the clearly opposing time courses (Table 1) no intergroup differences of these additional surrogate parameters were detectable in the post-hoc comparison.

The study has some limitations. At first, only a post-hoc comparison to another study [1] was performed. The availability of additional V_A_/Q as well as previously published data, a small but distinct hypothesis and animal care reasons may though justify this approach. Experimental setup, TIP peptide dosage and physiologic variables were accordingly predetermined.

Different model kinetics may interfere with these results. However, the histopathological correlates of LAV and OAI in pigs have been characterized in detail by our group: accordingly, a higher grade of epithelial necrosis occurs in the OAI model, though not necessarily a more severe gas exchange impairment [11]. OAI also interacts with the Na^+^-K^+^-ATPase on the basolateral side of type II alveolar cells and therefore prohibits the alveolar fluid clearance [12]. The LAV model, however, to some extent maintains the alveolar epithelial function [8,11,13]. A sustained improvement was found within three hours in the LAV model [1]. This analysis indicates that in contrast the time course of the OAI model yields no measurable effect after TIP inhalation. Furthermore, the V_A_/Q was significantly ameliorated in the LAV group in comparison to the OAI group. This different response pattern may possibly be related to the higher amount of epithelial destruction in the OAI model. Our hypothesis therefore was approved. These data vice versa can be interpreted as confirmation that in porcine models the enhancement of the alveolar fluid clearance rather than attenuation of hyperpermeability or inflammatory response is the main mechanism of TIP peptide inhalation. The apical side of the alveolar epithelium represents the TIP peptide’s main side of action [5,6]. Due to the ongoing clinical development of the TIP peptide these findings may have further relevancy: based on these considerations regarding the model characteristics the TIP peptide should require an intact alveolar epithelial function. Further research needs to determine, if this new approach can be effective in states of profound alveolar destruction like in longer established or persisting ARDS or only in early states that are primarily characterized by edematous respiratory failure and less alveolar necrosis. In summary, this report further elucidates the underlying mechanisms of the TIP peptide inhalation as potential pharmacotherapy in respiratory failure or ARDS.

## Abbreviations

ARDS: Acute respiratory distress syndrome; Cdyn: Dynamic compliance; CO: Cardiac output; etCO2: End-tidal carbon dioxide; EVLWI: Extravascular lung water index; FiO2: Fraction of inspired oxygen; LAV: Bronchoalveolar lavage/surfactant depletion; MAP: Mean arterial pressure; MPAP: Mean pulmonary artery pressure; OAI: Oleic acid injection; PaCO2: Arterial partial pressure of CO_2_; PaO2: Arterial partial pressure of oxygen; PEEP: Positive end-expiratory pressure; Pendinsp: End-inspiratory airway pressure; PVR: Pulmonary vascular resistance; RR: Respiratory rate; TIP: Synthetic peptide mimicking the lectin-like domain of TNF-α; VA/Q: Ventilation/perfusion-distribution; Vt: Tidal volume.

## Competing interests

The TIP peptide AP301 was provided by APEPTICO, Vienna, Austria. APEPTICO had no influence on the performance of the experiments, data analysis and interpretation or manuscript drafting.

## Authors’ contributions

EKH coordinated and supervised the experiments. EKH, AB, BD, KUK and SB conducted the experiments. EKH and BD performed the data analysis. EKH drafted the manuscript. KM and MD participated in the study design, supervision of laboratory, data analysis and revision of the manuscript. All authors edited and approved the final manuscript.
